# The drug-resistance to gefitinib in PTEN low expression cancer cells is reversed by irradiation in vitro

**DOI:** 10.1186/1756-9966-28-123

**Published:** 2009-09-01

**Authors:** Hong-Qing Zhuang, Jun Wang, Zhi-Yong Yuan, Lu-Jun Zhao, Ping Wang, Chang-Li Wang

**Affiliations:** 1 Department of Radiotherapy, Tianjin Medical University Cancer Institute and Hospital and Tianjin Key Laboratory of Cancer Prevention and Therapy, Tianjin, PR China; 2 Tianjin Lung Cancer Center, Tianjin, PR China

## Abstract

**Background:**

Despite of the recent success of EGFR inhibitory agents, the primary drug-resistant becomes a major challenge for EGFR inhibitor therapies. PTEN gene is an important positive regulatory factor for response to EGFR inhibitor therapy. Low-expression of PTEN is clearly one of the important reasons why tumor cells resisted to tyrosine kinase inhibitors.

**Methods:**

To investigate the drug-resistance reversal to gefitinb and the mechanism in PTEN low expression cells which radiated with X-rays in vitro, We demonstrated that H-157 lung cancer cells (low-expression of PTEN but phospho-EGFR overexpressed tumor cells) exposed to X-rays. The PTEN expressions and radiosensitizing effects of tyrosine kinase inhibitor before and after irradiation were observed. The cell-survival rates were evaluated by colony-forming assays. The cell apoptosis was investigated using FCM. The expressions of phospho-EGFR and PTEN were determined by Western blot analysis.

**Results:**

The results showed that the PTEN expressions were significantly enhanced by X-rays. Moreover, the cell growth curve and survival curve were down-regulated in the gefitinib-treated groups after irradiation. Meanwhile, the radiation-induced apoptosis of tumor cells was increased by inhibition of the EGFR through up-regulation of PTEN.

**Conclusion:**

These results suggested that PTEN gene is an important regulator on TKI inhibition, and the resistance to tyrosine kinase inhibitors might be reversed by irradiation in PTEN low expression cancer cells.

## Background

The EGFR is a receptor tyrosine kinase that regulates fundamental processes of cell growth and differentiation. Overexpression of EGFR and its ligands, were reported for various epithelial tumors in the 1980s [1,2] and generated interest in EGFR as a potential target for cancer therapy [3-9]. These efforts have been rewarded in recent years as ATP site-directed EGFR tyrosine kinase inhibitors has shown anti-tumor activity in subsets of patients with non-small cell lung cancer [10,11], squamous cell carcinomas of the head and neck [12], and selected other malignancies [13-17]. The current data from retrospectively analyzed clinical trials and preclinical models [18-23] suggested that monotherapy with EGFR kinase inhibitors is unlikely to be effective in PTEN-deficient tumors, even if they harbor activating EGFR mutations. This could potentially result in upfront resistance to EGFR inhibitors in highly PTEN-deficient tumors. However, there are little research on the drug-resistance of EGFR kinase inhibitors, and there is no suitable means for reversal of drug resistance in clinical practice until today. The data presented herein describe the resistance to tyrosine kinase inhibitors (TKI) reversed on PTEN low-expression cancer cells by irradiation in vitro. Our study may have potential impacts on the clinical applications of combining TKI with irradiation therapy in patients with cancers of primary drug-resistance to TKI.

## Materials and methods

### Reagents

Cell culture media was provided by Tianjin Medical University Cancer Institute (Jin-pu Yu, MD). Primary antibodies against phospho-EGFR and PTEN were obtained from Santa Cruz Biotechnology, Inc. (Santa Cruz, CA); Propidium Iodide (PI) and annexin V were purchased from Cell Signaling Company (Cell Signaling Technology, Beverly, MA). Gefitinib was generously provided by AstraZeneca (Zhen-yu You, Beijing). All the other materials were from Cancer Institute of our university.

### Cell lines and cell culture

The H-157 lung cancer cell line was kindly provided by Peking University Center for Human Disease Genomics. It was maintained in RPMI1640 supplemented with 20 mM HEPES (pH 7.4), 100 IU/mL penicillin, 100 mg/mL streptomycin, 4 mM glutamine and 10% heat-inactivated fetal bovine serum (Hangzhou Sijiqing Biological Engineering Materials Company, China) in a humidified atmosphere of 95% air and 5% CO2 at 37°C.

### Ionizing radiation treatment

Exponentially growing H-157 cells in a tissue-culture flask (75 cm^2^) were irradiated by an X-ray generator with a 1.0-mm aluminum filter at 200 kVp and 10 mA, at a dose of 1.953 Gy/min, which was determined using Fricke's chemical dosimeter. Then they were incubated for another 48 h at 37°C. Addition of Gefitinib was carried out at the same time when the treatment of irradiation was performed. Radiation was performed in the Tianjin Medical University Cancer hospital.

### Western blot analysis

To examine the phospho-EGFR and PTEN expression in H-157 cells, the protein was assayed by western blot analysis [24]. To determine whether irradiation causes an increase of PTEN expression, cells in culture were irradiated with 1, 2, 4, 6, 8 and 10 Gy. Following treatment, the cells were collected 3 h, 6 h, 9 h, and 12 h respectively. Total protein was extracted from H-157 cancer cell lines, resolved and analyzed by Western blotting. In brief, cells were washed with cold-phosphate buffered saline (PBS), scraped in RIPA buffer (100 mMTris, 5 mMEDTA, 5%NP40, pH8.0) containing protease inhibitors cocktail (Roche diagnostics, Mannheim, Germany) and allowed for at least 30 min on ice. Cells were subjected to further analysis by one freeze-thaw cycle and centrifuged at 14,000 g for 30 min at 4°C. Supernatants were carefully removed and protein concentrations were determined by Bio-Rad-DC protein estimation kit. Electrophoresis was performed on polyacrylamide gel (10%) using equal amounts of protein samples under reducing conditions. Resolved proteins were transferred to the PVDF membranes and probed with primary antibodies followed by incubation with corresponding horseradish peroxidase-conjugated secondary antibodies. Signal was detected with ECL electrochemiluminescence (ECL) Kit (Amersham Biosciences).

### Cell-growth analysis

Cell proliferation was determined by assessing the mitochondrial reduction of MTT. In brief, cells from the control and gefitinib-pre-treated groups were plated at 1 × 10^3 ^cells/well in 96-well plates containing 200 μL growth medium and allowed to attach for 24 h. The medium was removed, and the gefitinib-treated cells were quiesced for 2d in a medium supplemented with100, 500, 1000 nM gefitinib. The medium was changed on day 2 of the 4d experiment. At harvest, the medium was removed from the appropriate wells, replaced with 50 μL MTT solution (2.5 mg MTT/ml), and incubated for 4 h at 37°C. After incubation, the MTT solution was carefully aspirated and replaced with 150 μL DMSO. Cell growth was analyzed on a plate reader by using SoftMax program (Molecular Devices Corp., Menlo Park, CA). Experiments were performed in quadruplicate and repeated at least 3 times. At the same time, the antiproliferative effect of gefitinib on the growth profile in vitro of H-157 cell line was examined. Briefly, The cells were treated with different concentrations of gefitinib (100, 500, 1000 nM). The control and gefitinib-treated cells(1 × 10^4^/well) were seeded on 6-well plates, and proliferation was evaluated by cell counting using a hemocytometer after 1,2,3,4,5,6 and 7 days of culture. All measurements were done in triplicate [25,26]. The methods were also used to detect the antiproliferative effect of gefitinib after irradiation.

### Clonogenic survival

Clonogenic survival was the ability of cells to maintain clonogenic capacity and to form colonies. The treatment schedule for clone assay: there are 4 groups in the experiments (the control, irradiation and/or gefitinib-treated groups). Cells in culture were irradiated with 1, 2, 4, 6, 8 and 10 Gy, and the gefitinib concentration was 100 nM. Briefly, after exposure to radiation, cells were trypsinized, counted, and seeded for colony formation in 60 mm dishes at 200 to 10000 cells/dish. After incubation intervals of 14 to 21 days, colonies were stained with crystal violet and manually counted. Colonies consisting of 50 cells or more were scored. Experiments were done in triplicate [27].

### Detection of apoptotic cells by FCM

To examine whether enhancement of apoptosis in X-ray irradiated H-157 cells overexpressed with PTEN was associated with gefitinib, we tested the effects of EGFR inhibitors on the enhancement of apoptosis in H-157 cells with and without irradiation. Cells from the irradiation and combined with Gefitinib groups (100 nM) were exposed to the same radiation dosages (6 Gy). At 48 h after irradiation, the cells were harvested. And then, cells were trypsinized, counted, and washed twice with cold PBS. Cells used for tests were stained with propidium iodide (PI) and annexin V for 15 min in the dark and analyzed by fluorescence-activated cell sorting (FACS) using Coulter EPICS and ModFit software (Verity Software House, Topsham, MN). Each test was performed 3 times [28].

### Statistical analysis

Data was plotted as means ± standard deviation. Student's t test was used for comparisons. Differences were considered significant at P < 0.05.

## Results

### EGFR, PTEN expression of H-157 cells

**Figure 1:**
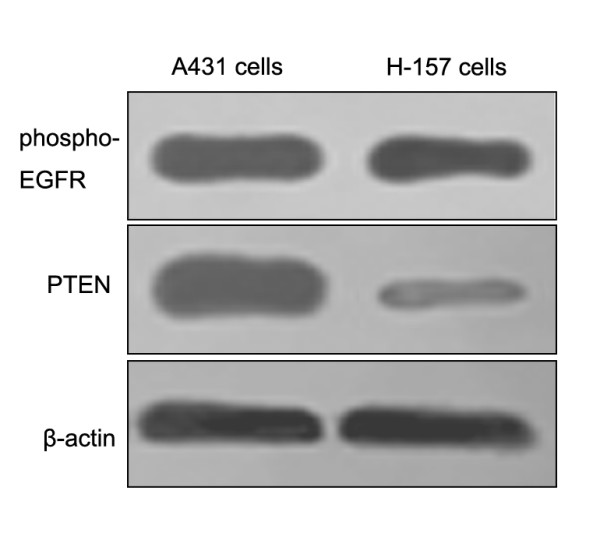
**Expressions of EGFR and PTEN in H-157 cells**. Western blots of EGFR (upper panel) and PTEN (lower panel) in H-157 cells. Both the EGFR and PTEN highly expressed cells, A431 cells, were taken as positive control.

It was reported that H-157 cells might be overexpression of phospho-EGFR and low-expression of PTEN [18]. In the present study, we confirmed the expression of phospho-EGFR and PTEN on the cells by western blotting. H-157 cells expressed high level of phospho-EGFR, but PTEN was low expressed. Both the phospho-EGFR and PTEN highly expressed cells, the A431 cells, were taken as positive control (Figure 1).

### Effects of gefitinib on H-157 cell growth

**Figure 2:**
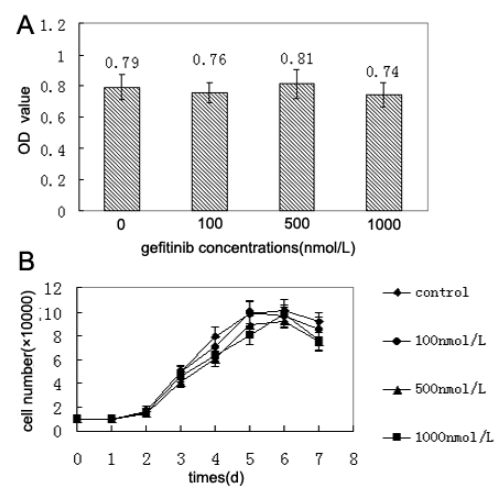
**Effects of gefitinib on H-157 cell growth in vitro**. (A)Cell proliferation was determined by assessing the mitochondrial reduction of MTT. Bars indicated means ± standard deviation of three independent experiments performed in triplicate (n = 9). Compared with untreated control cells, *P *> 0.05 were found in all of the treated groups. (B)Known numbers of single cells were plated into culture dishes in RPMI1640 containing 10% FBS and treated with gefitinib in several doses. Cells were then harvested by trypsinization and counted by a hemocytometer with trypan blue dye. Data points mean of triplicate samples. Data were expressed as means ± SE for three experiments. *P*> 0.05 vs. control group by Student's t-test was found in every treated group.

As shown in Figure 2, though different concentrations treatment produced no significant inhibition to H-157 cell growth. Cell counting was also used to assess the proliferative ability of gefitinib-treated cells. There was no significant difference in the growth rates between control cells and gefitinib-treated cells.

### Expression of PTEN in H-157 cells after irradiation treatment

**Figure 3:**
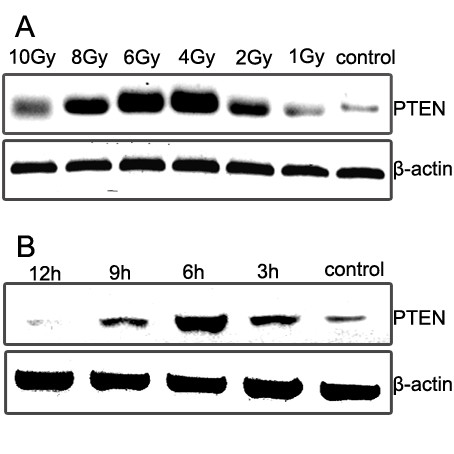
**Expression of PTEN in H-157 cells after irradiation treatment**. (A) The H-157 cells which exposed to 1, 2, 4, 6, 8, and 10 Gy of X-rays were analyzed as shown in right panel. After irradiation, the cells were incubated for 6 h, and then were examined. (B) After incubation of X-irradiated (6 Gy) cells for 3, 6, 9 and 12 h, the PTEN protein was examined by Western blotting. Irradiation Treatment was shown to increase PTEN levels in H-157 cell lines tested, and H-157 cells exposing to 4~6 Gy expressed major amounts of PTEN.

After different dosage radiation (0, 1, 2, 4, 6, 8, and 10 Gy), the PTEN expression increased in a time-dependent manner. The highest expression were observed in H-157 cells treated with 4~6 Gy irradiation. At the same time, we also measured that PTEN expression increased at 3 h and returned to baseline at 12 h after irradiation (Figure 3). Based on this, we concluded that 6 Gy was the best dosage for improving PTEN expression and the same time as treatment with irradiation was the optimal time for addition of gefitinib.

### Survival curve and cell growth curve of gefitinib-treated H-157 cells after irradiation

**Figure 4:**
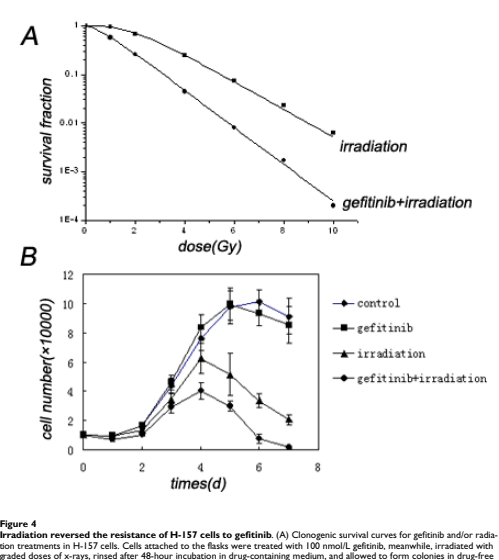
**Irradiation reversed the resistance of H-157 cells to gefitinib**. (A) Clonogenic survival curves for gefitinib and/or radiation treatments in H-157 cells. Cells attached to the flasks were treated with 100 nmol/L gefitinib, meanwhile, irradiated with graded doses of x-rays, rinsed after 48-hour incubation in drug-containing medium, and allowed to form colonies in drug-free medium. Surviving fractions for radiation + gefitinib were normalized by dividing by the surviving fraction for gefitinib only. Each test was performed 3 times. The radiation-enhancing (t = 7.65, P < 0.01) effect of gefitinib was comparable with that of gefitinib alone in H-157 cells. (B) Effects of gefitinib on H-157 cell growth after irradiation. There was no significant difference (t = 1.13, P > 0.05) in the growth rates between H-157 cells and gefitinib-treated cells as determined by cell counting, but the proliferative ability of the gefitinib and radiation treated cells was dramatically suppressed(t = 5.01, P < 0.05)in contrast with radiation-treated only.

The cloning efficiency of H-157 was between 60% and 90%. The survival curve of control and gefitinib-treated H-157 cells after irradiation was shown in Figure 4. The radiobiological parameters of H-157 cells treated with irradiation and gefitinib were D_0 _= 1.14, D_q _= 0.22, N = 1.57, while those of irradiation-treated H-157 cells were D_0 _= 1.51, D_q _= 0.88, N = 3.84. In the present study, SER (sensitive enhancement ratio) = D0 (irradiation+gefitinib group)/D_0 _(irradiation group) = 1.51/1.14 = 1.32. The SER in gefitinib-treated cells indicated that treatment with gefitinib significantly improved the biological effect of irradiation following PTEN high expressed. At the same time, the cell growth curve was also down-regulated by gefitinib after irradiation (Figure 4). The data presented herein suggested the resistance for gefitinib was reversed by irradiation in H-157cells.

### Gefitinib increased the radiation-induced apoptosis

**Figure 5:**
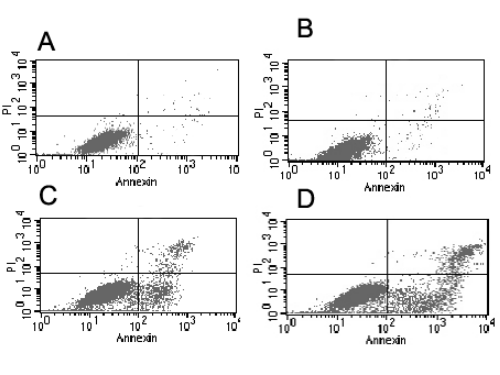
**Gefitinib-induced apoptosis in H-157 cells before and after irradiation**. Attached cells were exposed to 6 Gy irradiation and then treated with 100 nmol/L gefitnib. After additional 48-hour incubation in medium containing the drugs, the cells were harvested. The apoptotic index (AI) was measured using flow cytometry. (A) Control groups (AI: 1.36 ± 0.74%). (B) Apoptotic values after treatment with 100 nmol/L gefitinib alone (AI:3.58 ± 0.61%).(C) Radiation- induced apoptosis induction (14.26 ± 2.97%% of total cells) in H-157 cells.(D) Radiation combined with gefitinib induced apoptosis induction (23.58 ± 3.61% of total cells). Apoptotic values were normalized by subtracting control values; the normalized apoptotic values were used for statistical analyses. Experiments were done in triplicate. Combined drug treatments were shown to enhance radiation-induced apoptosis in H-157 cells (t = 19.91, P < 0.01), but no synergistic manner when compared with drug alone without radiation (t = 2.569, P > 0.05).

As shown in Figure 5. The early apoptosis rate among gefitinib-treated H-157 cells after 6 Gy irradiation was significantly higher than the cells with the same dosage of X-rays only. Whereas, no significant apoptotic changes were observed in unirradiated cells before and after gefitinib treated. Quantitative measurements of apoptotic cell death by FCM in H-157 cells sufficiently indicated that the radiation-induced overexpression of PTEN significantly enhanced gefitinib-induced apoptosis in comparison with that of the control (no irradiation).

## Discussion

The PI3K pathway is a critical effector of growth, proliferation, and survival pathways. PTEN serves as negative regulator of the phosphatidylinositol 3-kinase (PI3K) pathway by removing the third phosphate from the inositol ring of the second messenger PIP3 [29]. PTEN inactivation results in accumulation of PIP3 levels and persistent signaling through the serine/threonine kinase Akt/protein kinase B. PTEN loss could thus promote resistance to EGFR kinase inhibitors from downstream inhibition of the PI3K signaling pathway [30-35]. Consistent with this, increased levels of PTEN expression is considered as basic principle during reversal of resistance for tyrosine kinase inhibitors in patients with PTEN low-expression cancers. In order to improve PTEN expression, transfection by medium such as plasmid and virus is the common method in fundamental research [36-39]. However, in the clinical application, these methods have been unable to overcome its own errors and defects, for instance, transfection efficiency, distribution in vivo, and so on. Finding the effective methods which can reverse primary drug-resistance in PTEN low-expression cells has become the urgent task on targeted therapy in clinical practice.

As the drug resistant cell line for tyrosine kinase inhibitors due to low expression level of PTEN protein [18], H-157 cell line is the precondition of the experiment. Our results demonstrated that phospho-EGFR higher expression and low-expression of PTEN present in H-157 cells (Fig. 1), which are accorded with the reports of literatures [18]. We also observed that when the H-157 cells were exposed to gefitinib, no significant deference of cell growth curve (Fig. 2), and no increase in the apoptosis of cells was found (Fig. 5). In conclusion, findings from the present study indicated that gefitinib has no effective antiproliferative function for H-157 cells. As a safe and satisfied method in the clinical therapy of cancer, Irradiation is easy to obtained, and it has not the disadvantages such as maldistribution and low transfer efficiency of transfection. Compared with transfection, irradiation possesses incomparable superiority, and it is one of the best solutions of drug-resistant reversal for TKI therapy [40,41]. Meanwhile, our results indicated that exposure to gefitinib after radiation enhanced the radiosensitivity of H-157 cells (Fig. 4). Considering that an increase in the level of the PTEN expression, the negative regulator of the phosphatidylinositol 3-kinase (PI3K) pathway was increased after irradiation, and then the second messenger PIP3 levels decreased, further activation of the serine/threonine kinase Akt/protein kinase B declined. So, the cell proliferation decreased and the cell death increased [29]. Therefore, x-ray radiation might play a major role in the drug-resistance reversal.

It must point out that cross talk between different pathways makes miscellaneous of signal transductions in tumor cells. Up to now, it is still unclear that whether there exist other factors of PI3K pathway producing influence for tyrosine kinase inhibitors efficacy except for PTEN protein. Whether the factors are influenced by irradiation, and then made some effect on the drug-resistance further remains unknown. These all need further research on PI3K pathway.

## Conclusion

Our study provides a new idea for drug-resistance reversal of TKI targeted therapy, and makes a beneficial exploration on tyrosine kinase inhibitors combined with radiotherapy in TKI resistant tumors. We believe that, with the further development of fundamental research, we are looking forward to an increasing application prospect of tyrosine kinase inhibitors in clinical practice.

## List of abbreviations

**EGFR**: epidermal growth factor receptor; **PE**: plating efficiency; **SF**: survival fraction; **RBE**: relative biological effect; **D**_0_; mean lethal dose; **Dq**: quasithreshold dose; **N**: extrapolation number; **MTT**: methyl thiazolyl tetrazolium; **FCM**: flowcytometry; **TKI**: tyrosine kinase inhibitor; **PTEN**: phosphatase and tensin homolog on chromosome ten; **PIP3**: phosphatidyinositol-3, 4, 5- triphosphate; **PI3K**: phosphatidylinositol-3-kinase.

## Competing interests

The authors declare that they have no competing interests.

## Authors' contributions

HZ wrote the paper. ZY designed the research. JW, LZ, and PW carried out the molecular genetics studies. CW carried out the data analysis. All authors have read and approved the manuscript.
